# Presence of Extracellular DNA during Biofilm Formation by *Xanthomonas citri* subsp. *citri* Strains with Different Host Range

**DOI:** 10.1371/journal.pone.0156695

**Published:** 2016-06-01

**Authors:** Marta Sena-Vélez, Cristina Redondo, James H. Graham, Jaime Cubero

**Affiliations:** 1 Department of Plant Protection, Instituto Nacional de Investigación y Tecnología Agraria y Alimentaria (INIA), Madrid, Spain; 2 Citrus Research and Education Center (CREC), University of Florida, Lake Alfred, Florida, United States of America; Universidad de Costa Rica, COSTA RICA

## Abstract

*Xanthomonas citri* subsp. *citri* (*Xcc*) A strain causes citrus bacterial canker, a serious leaf, fruit and stem spotting disease of several *Citrus* species. *X*. *alfalfae* subsp. *citrumelonis* (*Xac*) is the cause of citrus bacterial spot, a minor disease of citrus nursery plants and *X*. *campestris* pv. *campestris* (*Xc*) is a systemic pathogen that causes black rot of cabbage. *Xanthomonas* spp. form biofilms *in planta* that facilitate the host infection process. Herein, the role of extracellular DNA (eDNA) was evaluated in the formation and stabilization of the biofilm matrix at different stages of biofilm development. Fluorescence and light microscopy, as well as DNAse treatments, were used to determine the presence of eDNA in biofilms and bacterial cultures. DNAse treatments of *Xcc* strains and *Xac* reduced biofilm formation at the initial stage of development, as well as disrupted preformed biofilm. By comparison, no significant effect of the DNAse was detected for biofilm formation by *Xc*. DNAse effects on biofilm formation or disruption varied among *Xcc* strains and *Xanthomonas* species which suggest different roles for eDNA. Variation in the structure of fibers containing eDNA in biofilms, bacterial cultures, and in twitching motility was also visualized by microscopy. The proposed roles for eDNA are as an adhesin in the early stages of biofilm formation, as an structural component of mature bacterial aggregates, and twitching motility structures.

## Introduction

Biofilms adhere bacteria to surfaces within a tridimensional structure that protects the bacterium against antibiotics and abiotic stresses [1–4]. Most biofilms are composed of 10% bacteria and 90% extracellular polymeric matrix [5]. The extracellular matrix provides resistance to temperature, pH and other deleterious conditions and also confers mechanical stability. Biofilms are composed of water, proteins, exopolysaccharide, lipopolysaccharide, lipids, surfactants and extracellular DNA (eDNA) [5,6].

eDNA is an important component of the extracellular matrix in biofilms for several Gram-positive, Gram-negative bacteria [7–12] and archaea [13]. The presence of eDNA in the extracellular matrix was described in 1956 [14] and its importance in biofilm formation was first demonstrated for *Pseudomonas aeruginosa* in the early stages of bacterial adhesion [10]. Meanwhile, in clinical isolates of the same species, eDNA was found to be important for stability of the biofilm [11]. In *P*. *aeruginosa* biofilms, eDNA coats the surface like a net in which bacteria are attached, and may also form a mushroom-like cap over the outer surface of the aggregate [15]. Besides biofilm formation, eDNA contributes to bacterial resistance to antibiotics, e.g., a *Bacillus cereus* mutant, that did not produce eDNA, was more susceptible to actinomycin D than the wild type [9]. eDNA has also been demonstrated to act as nutrient source of phosphorus, nitrogen and carbon. DNAse in *P*. *aeruginosa* secreted via the Type II Secretion System is related with nutrient acquisition, biofilm dispersal and horizontal gene transfer [16–18]. At high concentrations, eDNA produces antimicrobial activity by chelating cations and destabilizing the bacterial outer membrane through lipopolysaccharide modification [19]. These findings identify eDNA as an important component of the extracellular bacterial matrix that plays a role in several processes related to bacterial colonization and virulence.

*Xanthomonas citri* subsp. *citri* (*Xcc*) (formerly *X*. *axonopodis* pv. citri) is the causal agent of Asiatic citrus bacterial canker (CBC), a serious disease of many citrus species [20,21]. *X*. *alfalfae* subsp. *citrumelonis* (*Xac*) is the cause of citrus bacterial spot (CBS), a minor foliar disease of young citrus plants in nurseries [22]. *Xcc* produces necrotic lesions on leaves, twigs and fruits that reduces fruit quality and marketability and restricts commercialization of plants and fruits in markets free of CBC [20]. *Xcc* A strain type is by far the most severe and widespread CBC pathogen and affects the widest range of *Citrus* species. Within *Xcc* A strains, two narrow host range variants have been described, *Xcc* A* and A^w^ from Southwest Asia and Florida, respectively, that cause CBC on Mexican lime (*Citrus aurantifolia*) [23–27]. *X*. *campestris* pv. campestris (*Xc*) is the causal agent of black rot, one of the most important diseases of crucifers worldwide. Black rot is a systemic vascular disease that causes symptoms including marginal leaf chlorosis, necrosis, darkening of leaf veins and vascular tissue within the stem [28,29].

*Xcc* and *Xc* produce biofilm to facilitate the infection process [3,30,31]. Several mechanisms in *Xcc* contribute to biofilm formation during the host-pathogen interaction: adhesins, the type III Secretion System, lipopolysaccharide, exopolysaccharide, type IV pili associated to twitching motility and chemotaxis [31–41]. Furthermore, biofilm formation *in planta* is related to the different host range of *Xcc* strains [42].

The roles that eDNA play in attachment and biofilm formation are not widely characterized for plant-bacterial interactions. Indeed, presence of eDNA in the extracellular matrix and in the different phases of biofilm formation have not been studied for *Xanthomonas* spp. Herein, we report on the contribution of eDNA to biofilm formation and the related twitching motility for several *Xcc* strains with different host range and ability to form biofilms, and compare these features with those of *Xc*, a systemic pathogen of cabbage, and *Xac*, a citrus pathogen able to infect and produce biofilm on citrus leaves [20,22,42]. In these studies, presence of eDNA was corroborated after DNase treatments and visualized by fluorescence staining of bacterial cultures, biofilms and bacteria undergoing twitching motility associated with bacterial aggregation.

## Results

### Detection of eDNA in *Xanthomonas citri* subsp. *citri* strains

**Fig 1:**
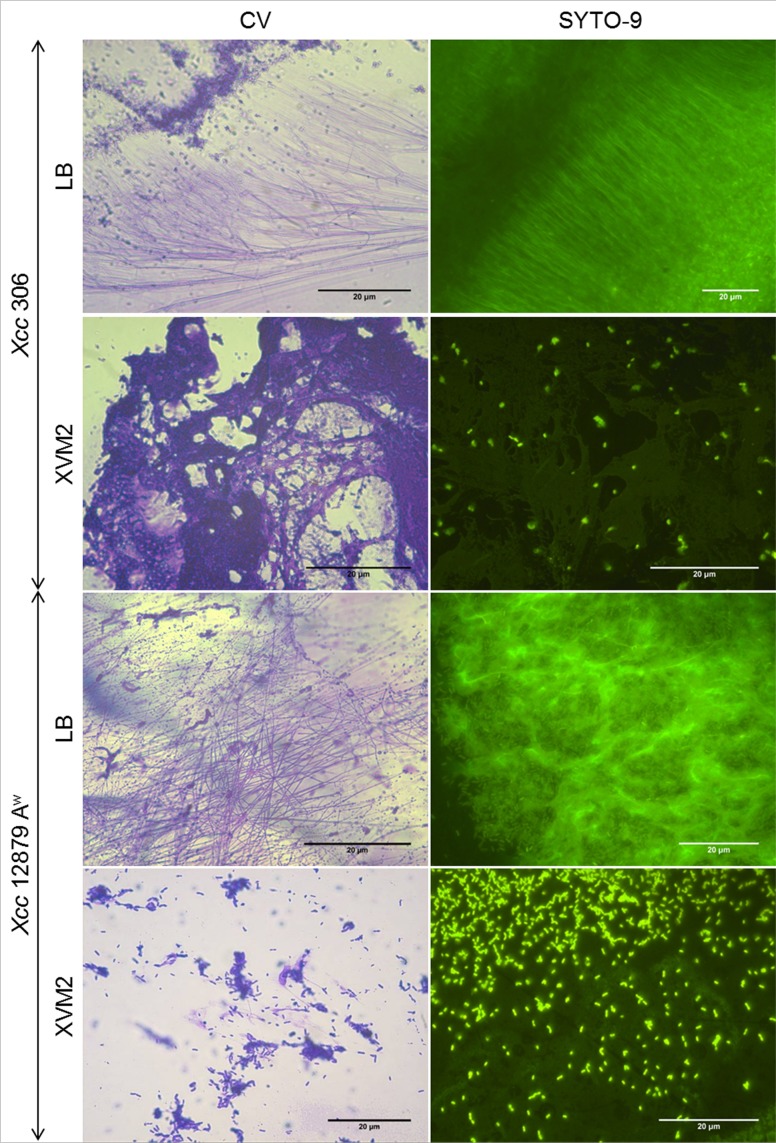
Presence of eDNA at the early stages of biofilm formation in *Xcc*. Representative light (crystal violet, CV staining) and fluorescence (SYTO-9 staining) images of 72 h static cultures on LB or XVM2 media. Fibers were observed after both staining for strains *Xcc* 306 and *Xcc* 12879 A^w^ at the early stages of biofilm formation. Fibers interconnected cells at different stages of aggregation, from one to several cells are shown. In XVM2 medium, fibers were thicker and more uniform after staining with CV and SYTO-9. eDNA in XVM2 medium appeared to cover the surface like a sheet in contrast to individual fibers produced in LB medium.

**Fig 2:**
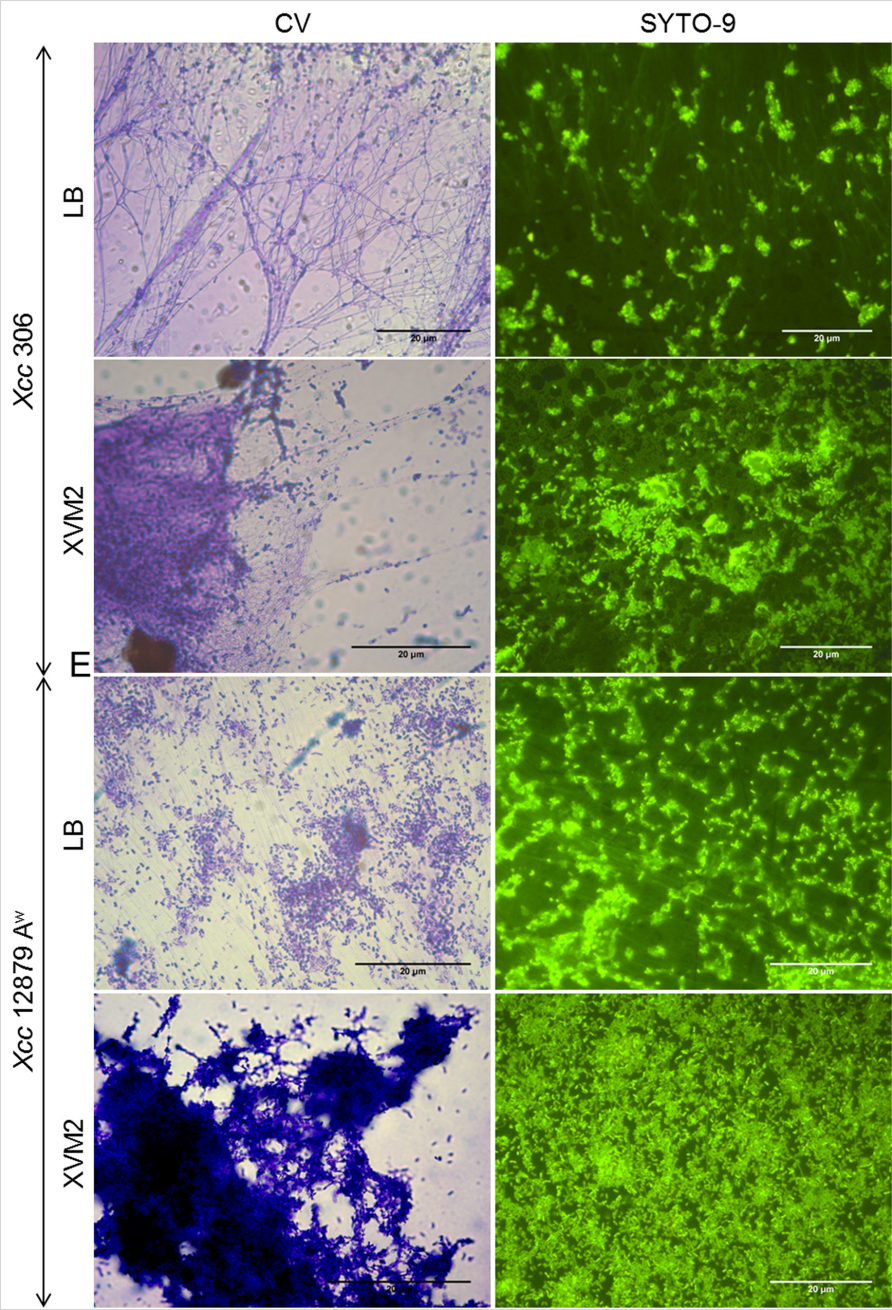
Presence of eDNA in preformed biofilms of *Xcc*. Representative light (CV staining) and fluorescence (SYTO-9 staining) images of mature biofilms on LB or XVM2 media. Both *Xcc* 306 and *Xcc* 12879 A^w^ strains were more aggregated in XVM2 than LB. A high level of aggregation for strain *Xcc* 12879 A^w^ in XVM2 made it difficult to observe eDNA fibers in aggregates. In XVM2, strain *Xcc* 306 was less aggregated and CV and SYTO-9 staining revealed eDNA surrounding the cells. In LB both staining revealed long fibers interconnecting aggregates.

**Fig 3:**
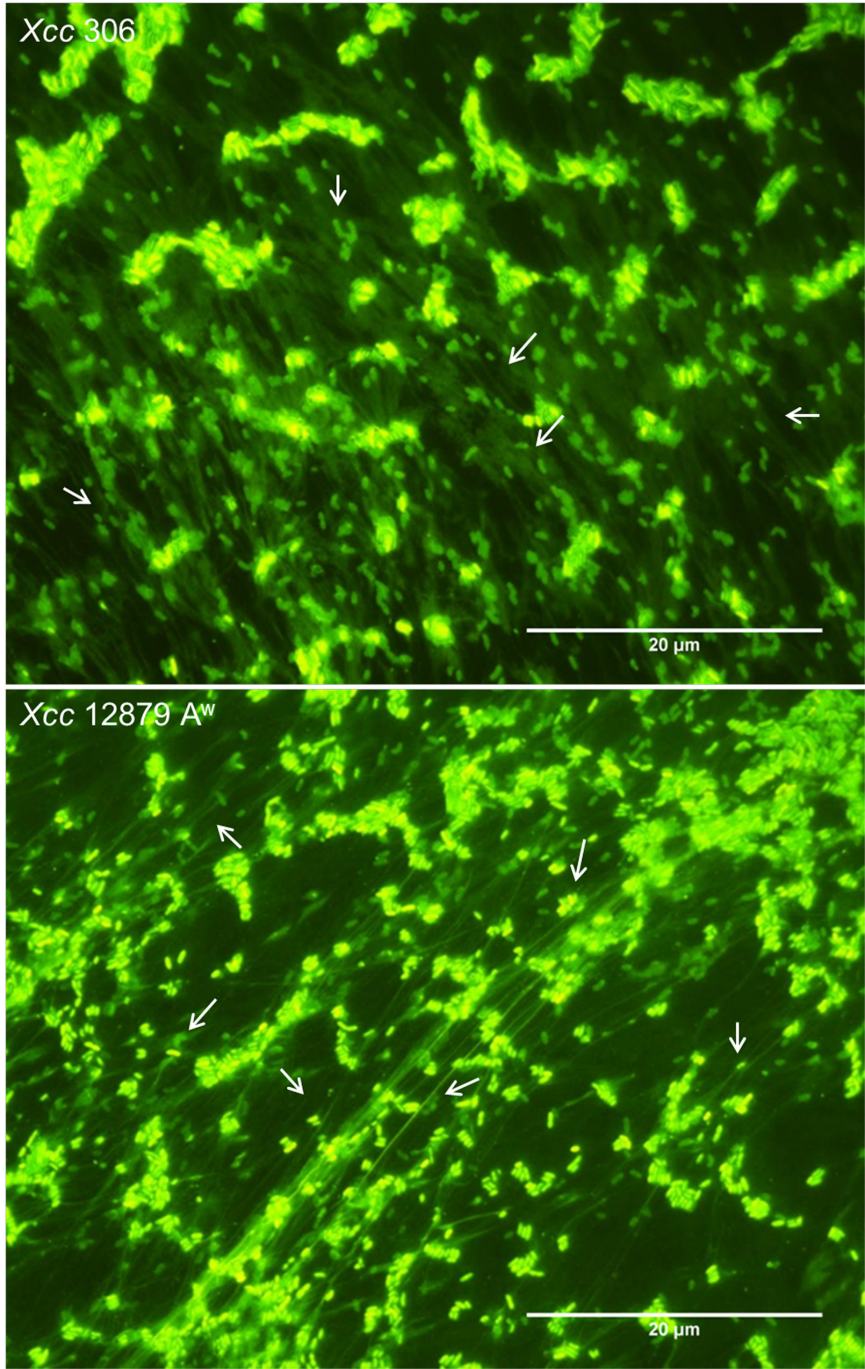
eDNA in exponential growth phase of *Xcc*. Representative fluorescence microscopy images of *Xcc* 306 and *Xcc* 12879 A^w^ in LB broth at exponential growth phase stained with SYTO-9. A high level of eDNA fibers (marked with white arrows) were produced by both strains. Bacillary shape bacteria are distinguishable from the fibers that appeared to connect cells over a long distance. *Xcc* A^w^ 12879 fibers were well developed while strain *Xcc* 306 fibers were more diffuse.

**Fig 4:**
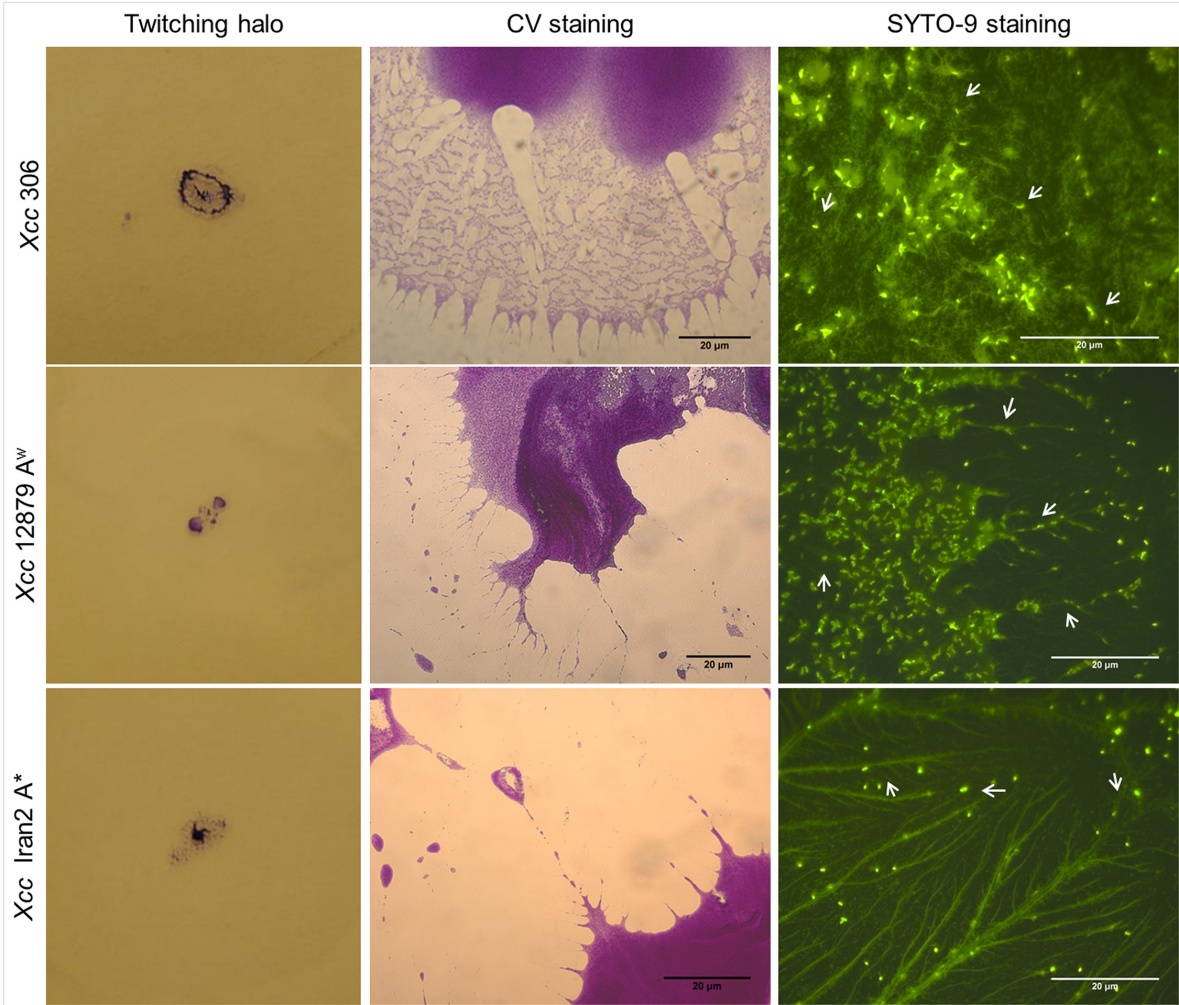
Presence of eDNA in *Xcc* twitching motility. Twitching halos from *Xcc* 306, 12879 A^w^ and Iran2 A* and light and fluorescence microscopy of twitching halos stained with CV or SYTO-9. Twitching motility was observed between the plate surface and the medium. CV and SYTO-9 staining of strains revealed similar twitching structures. Fibers observed after CV staining appeared to have a high eDNA content. Twitching fibres were longer for *Xcc* 12879 A^w^ and Iran2 A* than for *Xcc* 306. Some fibers are marked with white arrows to highlight diference from bacillary shape bacteria associated.

Presence of eDNA during biofilm formation was confirmed by SYTO-9 staining of bacterial cultures at different stages (Figs 1 and 2, right side). Furthermore, eDNA was observed in bacterial cultures at the exponential growth phase (Fig 3), after plate growth (S1 Fig) and in twitching motility assays (Fig 4, right side). In addition, CV staining of the same cultures (Figs 1, 2 and 4) revealed similar extracellular structures to those observed with SYTO-9 and fluorescence microcopy. Microscopy observations of GFP tagged cells stained with propidium iodide (S1 Fig) indicated that fiber fluorescence is due to the presence of eDNA and not caused by bacterial cell aggregation. In all assays bacterial cells in a bacillary shape were distinguishable from the containing DNA fibers. These results confirm that most of the extracellular fibers have a high eDNA content.

Microscopic observations of biofilm development were in agreement with previously reported studies, since *Xcc* aggregation was higher in minimal than nutrient rich medium [31,42]. Less bacterial aggregation and biofilm formation for both wide and narrow host range strains of *Xcc*, *Xcc* 306 and *Xcc* 12879 A^w^, were observed in LB medium than in XVM2 (Figs 1 and 2). The difference in biofilm formation between culture media was more marked for *Xcc* 12879 A^w^ than *Xcc* 306 [42].

eDNA appeared to be associated with long fibers of variable thickness and to an amorphous mass surrounding bacterial cells (Figs 1, 2, 3, 4 and S1 Fig). Fibers containing eDNA were observed in static cultures after 72 h (early stage of biofilm formation) as well as in mature biofilms (Figs 1 and 2). For strains *Xcc* 306 and *Xcc* 12879 A^w^ on LB and XVM2 media, the fibers appeared to interconnect bacterial cells within the aggregates. However fibers in LB medium were longer, thinner and less connective than in XVM2 medium. The short and branched filaments in XVM2 appeared to provide higher bacterial connectivity and aggregate stability. Differences in the development of eDNA fibers were also observed between *Xcc* 306 and *Xcc* 12879 A^w^ in exponential growth phase (Fig 3) and plate growth (S1 Fig) in LB medium. Similar results were observed in the twitching motility assay (Fig 4); fibers produced by strains *Xcc* 12879 A^w^ and Iran 2 A* were longer than the fibers produced by *Xcc* 306 that correlated to the different phenotype of the twitching colonies demonstrated for narrow and wide host range strains.

### Importance of eDNA in *Xanthomonas* biofilm formation

**Fig 5:**
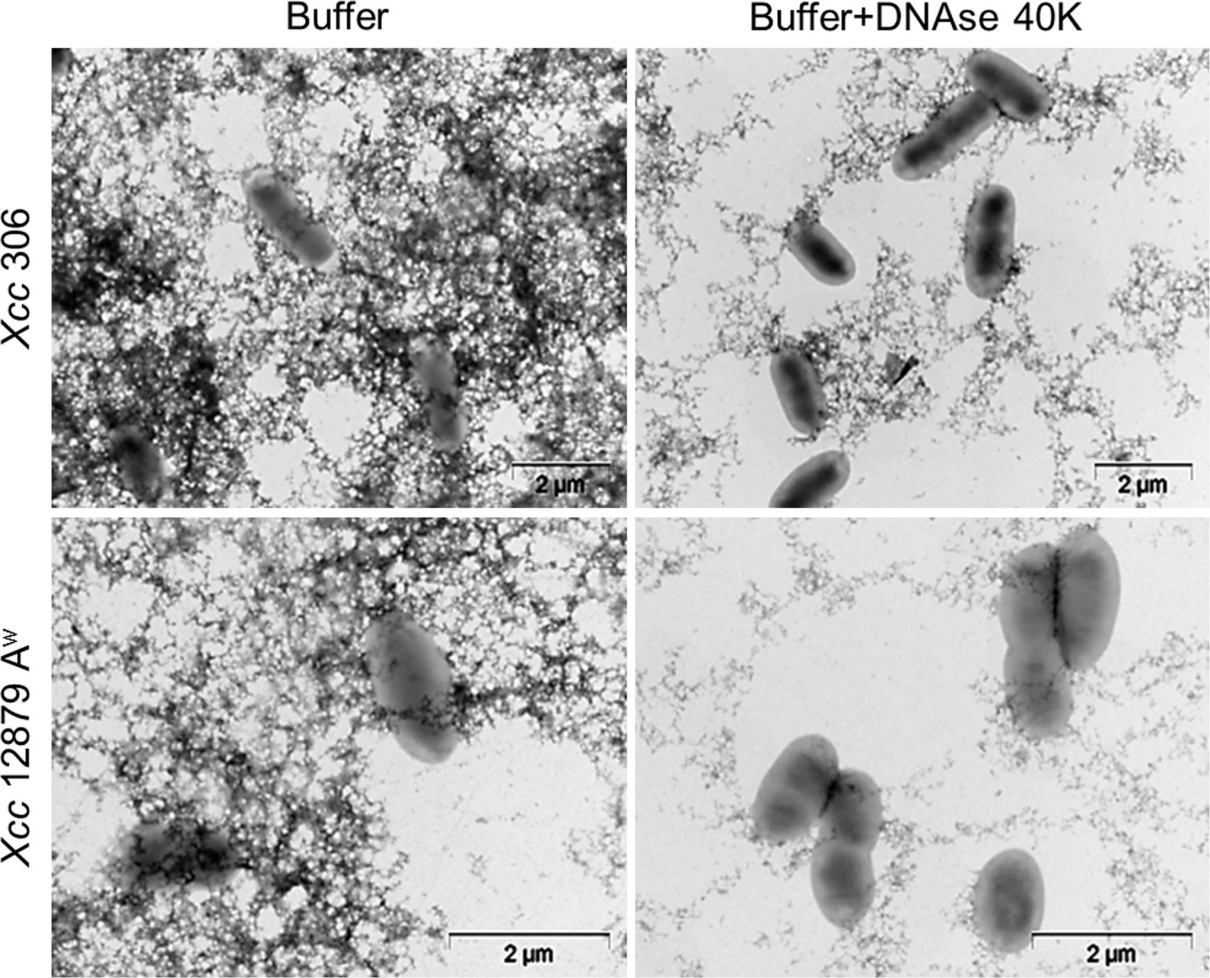
Effect of DNAse in the extracellular matrix of *Xcc*. Transmission Electron Microscopy of *Xcc* 306 and 12879 A^w^ strains in exponential growth phase, after DNAse and no-DNAse (Buffer) treatments. DNAse treated bacteria showed less extracellular structures than de buffer control. The remaining structures observed after treatment could be associated to extracellular proteins. Bacterial cell division is shown under DNAse treatment.

The role of eDNA in biofilm formation was estimated by measuring biofilm formation after DNAse I treatment at 0, 24, 48 or 72 hours post seeding (hps) by staining biofilms with crystal violet (CV) as described in Material and Methods. Transmission electron microscopy of *Xcc* cultures treated with DNAse showed reduction of extracellular fibers (Fig 5), confirming the DNAse activity and therefore the eDNA content. The bacterial population was not reduced by DNAse treatment (S2 Fig) and cell division in LB medium was shown under DNAse treatment (Fig 5).

**Fig 6:**
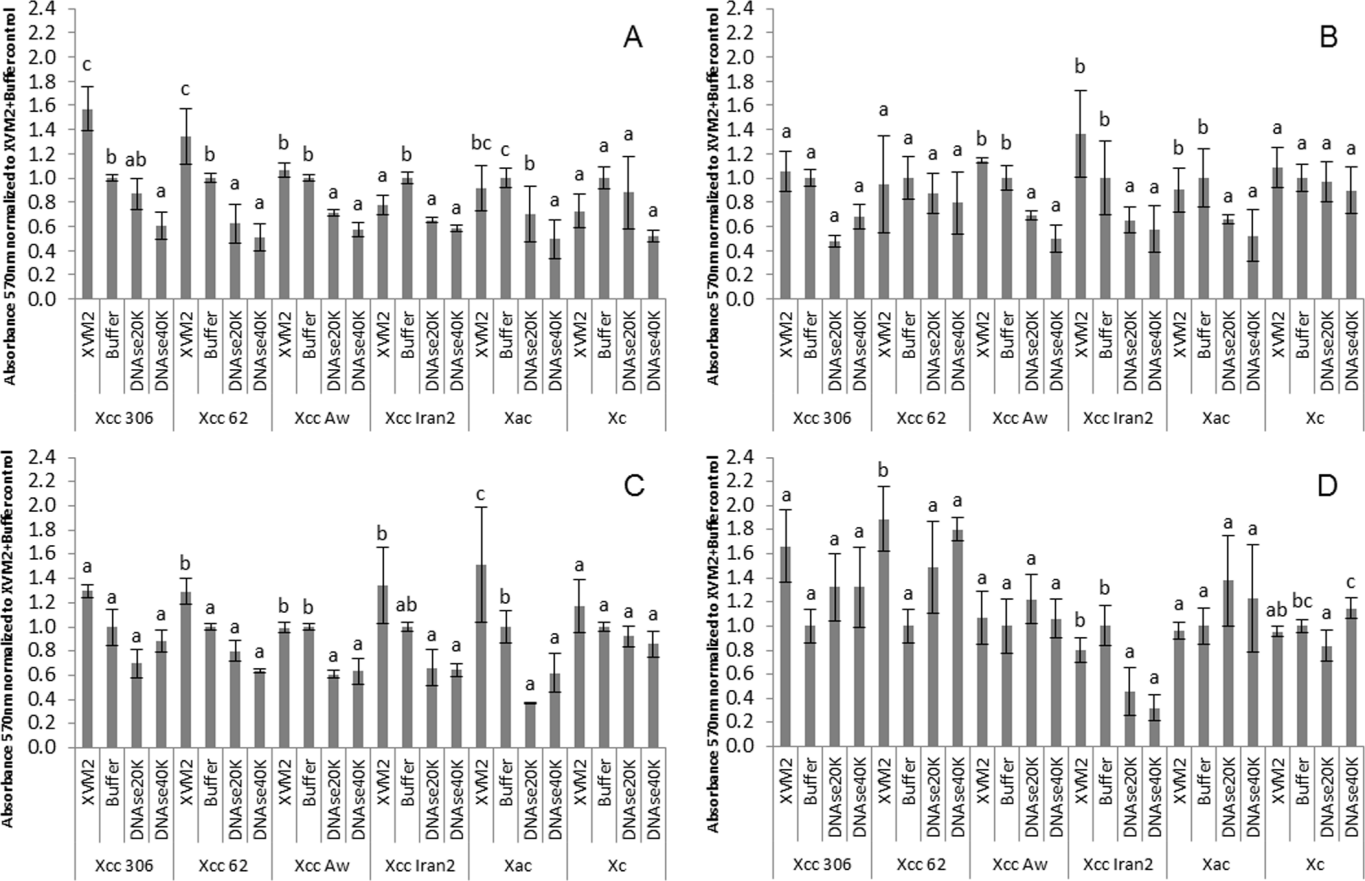
Effect of DNAse I during biofilm formation in *Xanthomonas*. Biofilm formation after treatment with DNAse I at different times after bacterial seeded with different *Xanthomonas* species and strains (see Table 1). A, DNAse added at 0 h post seeding (hps); B, DNAse added at 24 hps; C, DNAse added at 48 hps; and D, DNAse added at 72 hps. The absorbance values were normalized to the control XVM2 plus buffer in order to compare the response for different strains. Error bars represent the standard deviation. Graphs are a representative assay of at least three assays with three replicates per assay. Statistical analyses were performed using STATGRAPHICS Plus, version 5.1 (Copyright Manugistics Inc.).

Biofilm formation was compared in XVM2 medium, XVM2 medium plus buffer, and XVM2 medium plus buffer and DNAse I at 20 or 40 Kunitz mL^-1^. In most cases, reduction of biofilm formation was dependent on the timing of DNAse I treatment, but was not dependent on DNAse I concentration (Fig 6).

Reduction of biofilm was significant (*p<0*.*05*) for citrus xanthomonads and DNAse I application time. In contrast, DNAse I did not produce a statistically significant reduction of biofilm formation by *Xc* 1609 strain (*p>0*.*05*). Citrus *Xanthomonas* strains varied widely in response to DNAse I (Fig 6). In wide host range strains *Xcc* 306 and *Xcc* 62 biofilm formation was statistically reduced (*p<0*.*05*) when DNAse I was added at 0 hps but no reduction was observed at longer exposure times (*p>0*.*05*). For narrow host range strains, *Xcc* 12879 A^w^ and Iran2 A*, and *Xac* F1 biofilm formation was reduced by DNAse I treatmentsat 0, 24 and 48 hps. Furthermore, biofilm produced by the Iran2 A* strain was reduced at 72 hps after DNAse I treatment.

### Importance of eDNA in preformed biofilm

**Fig 7:**
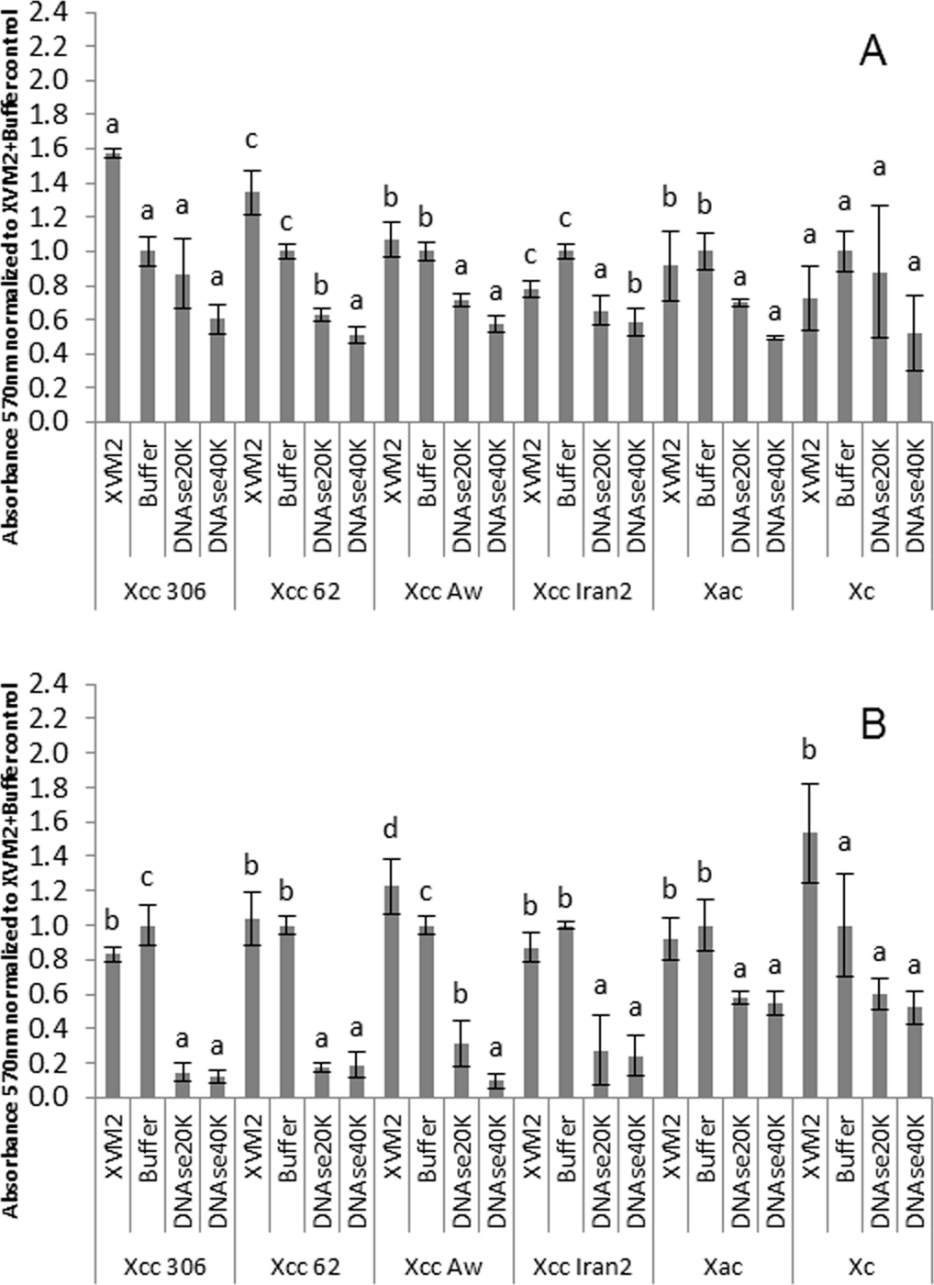
*Xanthomonas* biofilm rupture by DNAse I. A, DNAse treatment of 1 h duration. B, DNAse treatment for an overnight period. The absorbance values were normalized to the control XVM2 plus buffer in order to compare the response for different strains. Error bars represents the standard deviation. Graphs are a representative assay of at least three assays with three replicates per assay

To evaluate the role of eDNA in mature biofilms, DNAse I treatments were performed for 1 h or overnight. Biofilm disruption was observed after both incubation periods but overnight treatment had greater effect (Fig 7). DNAse I treatment for 1 h did not affect strains *Xcc* 306 and *Xc* 1609, but significantly (*p<0*.*05*) ruptured biofilm for strains *Xcc* 62, 12789 A^**w**^, Iran 2 A* and *Xac* F1 (Fig 7A). Overnight DNAse treatment of *Xcc* strains affetcted up to 80–90% of the biofilm and no differences were observed between wide and narrow host range *Xcc* strains (Fig 7B). Similar treatment did not significantly (*p>0*.*05*) disrupt biofilm of *Xc* 1609 compared to the buffer control. In summary, biofilm disruption by DNAse I was greater for *Xcc* strains than *Xac* F1 or *Xc* 1609.

## Discussion

Biofilm formation is an important virulence factor in *Xcc* [3,31]. Furthermore several differences in biofilm structures and biofilm formation have been observed between wide and narrow host range strains of *Xcc* which was related to their ability to infect the host [42]. The variation in function of eDNA in biofilm formation between wide and narrow *Xcc* strains could also be associated with the ability to form biofilms and their structure.

Extracellular DNA (eDNA) is an important component of the biofilm matrix in several bacteria, playing roles in adhesion, bacterial aggregate stability, nutrition, protection and gene exchange [9,10,16–19]. In this study, eDNA was detected either at different stages of the biofilm or in cultures and during twitching motility in citrus *Xanthomonas* strains with different host range. The presence of eDNA in biofilms for citrus *Xanthomonas* was determined and compared to *Xc*.

eDNA in *Xcc* has been observed in fibers interconnecting bacterial cells, possibly associated with other extracellular components including proteins and polysaccharides. Amorphous eDNA was also observed which is possibly eDNA that had been recently secreted, but not processed into fibers. Fibers were observed during biofilm formation in LB and XVM2 media, however those fibers formed in XVM2 appeared to be thicker and more uniform than those in LB medium, which is consistent with previous reports of higher biofilm formation in XVM2 medium. In previous reports [31,42] biofilm formation varied between *Xcc* 306 and 12879 A^w^, especially in LB medium. In XVM2, a culture medium that mimics apoplastic conditions, several pathogenicity factors are expressed for *Xcc* strains [26,43] and among them, eDNA could be considered an important virulence component in the biofilm for plant infection.

In *P*. *aeruginosa*, eDNA is a factor associated with twitching motility [44]. Twitching is enabled through the extension and retraction of the type IV pilus [45] which binds to DNA [46,47]. Herein, SYTO-9 staining of twitching assay plates revealed the presence of fibers containing eDNA during this type of movement recently been described for *Xcc* [32,48]. Moreover, twitching structures varied among narrow and wide host range strains of *Xcc* as also observed for biofilm formation on plant surfaces [42]. Twitching fibers of narrow host range strains were longer than those of the wide host range strains which had more interconnecting fibers, this may be related with the variable twitching halo shape between wide and narrow host range *Xcc* strains. Interestingly, *Xcc* cells were always connected by fibers containing eDNA. In *P*. *aeruginosa*, eDNA along with surfactants, were secreted onto the culture plate surface as the bacterium was undergoing motility [44]. In our study, *Xcc* appeared to track the eDNA in fibers possibly through the type IV pilus, since no surfactant activity was detected for *Xcc* (results not shown). Our hypothesis is that eDNA is associated with fibers on the plant surface in the initial stage of the colonization process, that involves motility and bacterial aggregation. Bacteria twitch across the surface by binding the type IV pilus with eDNA fibers deposited on the surface.

Biofilms treatments with DNAse confirmed the role of eDNA in biofilm formation in citrus related *Xanthomonas*. In many cases, DNAse treatment reduced biofilm formation depending on the strain and the biofilm stage. DNAse did not cause mortality or reduce bacterial growth rate, but was limited to the effect on biofilm formation (S2 Fig). When DNAse treatment terminated, bacterial attachment to the surfaces and aggregation resumed thereafter.

eDNA has been described as an adhesin in *B*. *cereus* [9], *Staphylococcus epidermis*, *S*. *aureus*, *S*. *mutants* [8] and *P*. *aeruginosa* [10]. Herein, similar eDNA properties were demonstrated for citrus strains of *Xanthomonas*. eDNA appeared to be important for the early stages of attachment by wide host range *Xcc* strains, whereas narrow host range strains required eDNA for biofilm development over an extended period, concurring with their higher requirements for biofilm formation. *In planta*, at 24 hpi, wide host range *Xcc* strains produced more complex structures in biofilms than the narrow host range *Xcc* strains and *Xac* strain which aggregated by producing long fibers [42]. The less interconnected fibers of the narrow host range *Xcc* strains and *Xac* strain may have rendered them more susceptible to DNAse disruption of aggregated cells and altered their biofilm formation. Other extracellular structures such as fimbria or flagella could influence biofilm formation and those structures may possibly be associated with eDNA since most of the fibers observed with CV stain were also stained with SYTO-9.

The role of eDNA in the stability of biofilms is important for bacteria including *E*. *coli*, *B*. *subtilis* [49], *S*. *mutants* [50], some strains of *P*. *aeruginosa* [11] and, as this study reports, strains of *Xcc* and *Xac*. Mature biofilms of citrus strains, but not the crucifer strain *Xc* 1609, were disrupted when exposed to DNAse. This observation suggests that the systemic pathogen *Xc* has a low dependence on eDNA for biofilm formation, surface attachment and stability of the biofilm or that eDNA is associated with other structures, making them inaccessible to DNAse I. Our hypothesis is that *Xc* moves systemically and aggregation would not be occurring after the initial stage of the plant colonization [51]. Hence, the role of eDNA in the different bacteria, depends on the structure and function of biofilm in plant colonization process in the *Xanthomonas* strain-host interaction.

Biofilm has been described as major factor for *Xcc* virulence and survival in citrus [3,31]. Several factors influence biofilm for wide host range *Xcc* strains. Mutants in the type IV pili produced biofilm but less structured than that produced by the wild type strain [32]. Non-fimbrilar adhesins have been described in the initial attachment of *Xcc* to abiotic surfaces [33]. Our findings provide a clue to a plausible role of eDNA acting in early stages of the biofilm as an adhesin in conjunction probably with other adhesins. Many studies describe the disruption of eDNA in animal bacterial pathogens, for examples, use of recombinant DNAse as a treatment against *P*. *aeruginosa* for cystic fibrosis patients [52] and extracellular DNAse (NucB) from *B*. *licheniformis* to inhibit and break bacterial biofilms [53]. Although biofilm formation and motility are well-described virulence factors for many plant pathogenic bacteria, occurrence and function of eDNA in plant pathogens in these processes is less well known. The determination of roles of eDNA in plant pathogenesis may lead to a better understanding of the plant-pathogen interaction and perhaps the development of tactics to disrupt eDNA to effect bacterial disease control.

## Material and Methods

### Bacterial strains, culture media and growth conditions

Bacterial strains used in this study and their natural hosts are listed in Table 1. Two wide host range strains of *Xcc* A, and two narrow host range strains, A* and A^w^, were evaluated along with *X*. *alfalfa*e subsp. *citrumelonis* and *X*. *campestris* pv. campestris. Bacterial strains were routinely grown in Luria Bertani (LB) broth (10 g tryptone, 5 g yeast extract and 5 g sodium chloride per litre) or on LB plates (1.5% bacteriological agar) at 27°C for 48 h.

**Table 1:** Strains of *Xanthomonas* spp. used in the study.

**Strain**	Taxon, or disease and CBC type	Natural Host
*Xcc* 306	*Xanthomonas citri* subsp. *citri*, CBC^a^ A	*Citrus spp*.
*Xcc* 62	*Xanthomonas citri* subsp. *citri*, CBC A	*Citrus spp*.
*Xcc* Iran *2*	*Xanthomonas citri* subsp. *citri*, CBC A*	*C*. *aurantifolia*
*Xcc* 12879 A^w^	*Xanthomonas citri* subsp. *citri*, CBC A^w^	*C*. *aurantifolia*
*Xac* F1	*Xanthomonas alfalfae* subsp. *citrumelonis*, CBS^b^	*Citrus spp*.
*Xc 1609*	*Xanthomonas campestris* pv. campestris CBR^c^	Cabbage
306 pUFZ75^d^	*Xanthomonas citri* subsp. *citri*, CBC A	
12879 pUFZ75^e^	*Xanthomonas citri* subsp. *citri*, CBC A^w^	

^a^Citrus Bacterial Canker (CBC)

^b^Citrus Bacterial Spot (CBS) and

^c^Crucifer Black Rot (CBR).

^d,e^GFP transformed strains with pUFZ75 plasmid [3,42] from *Xcc* 306 and *Xcc* 12879 *A*^*w*^, respectively.

In addition and to discriminate bacterial cells from eDNA contained in fibres, GFP tagged strains of *Xcc* 306 and *Xcc* 12879 A^w^ were used [3,42]. GFP expressing strains were grown on LB broth or plates with the addition of kanamycin at 50 μl mL^-1^ (LB+K).

### eDNA staining

To detect eDNA, samples were stained with SYTO-9 from the Live/Dead Bacterial viability kit (Molecular Probes Europe BV; Leiden, The Netherlands) and observed with fluorescence microscopy. SYTO-9 stains DNA and is also able to penetrate bacterial membranes [54,55]. Crystal Violet (CV) staining was also performed to visualize extracellular structures as previously described for flagella visualization of *Xcc* [56]. To determinate the presence of eDNA in biofilms, a *Xcc* 306 and *Xcc* 12879 A^w^ colony was taken and seeded into 5 mL LB medium for an overnight period, a 60 μL aliquot was then seeded in 30 mL of LB medium an incubated for an overnight period. Finally bacteria in exponential growth phase (0.5–0.6 ODs) were washed twice in 10mM MgCl_2_ and diluted to a concentration of 10^8^ cfu mL^-1^ in 25 mL of XVM2 (20 mm NaCl, 10 mm (NH_4_)_2_SO_4_, 5 mm MgSO_4_, 1 mm CaCl_2_, 0.16 mm KH_2_PO_4_, 0.32 mm K_2_HPO_4_, 0.01 mm FeSO_4_, 10 mm fructose, 10 mm sucrose, 0.03% casamino acid) [43,57] or LB media, and incubated without shaking at 27°C in a 50 mL borosilicate flask. In order to detect eDNA at the early stages of biofilm, bacteria growing and deposited on bottom of the flask were collected at 72 hours post seeding (hps) and stained either with SYTO-9 for 20 min in the dark or with CV. For mature biofilm eDNA detection, after 72 hps of static growth in 50 mL borosilicate flask, the liquid medium was decanted and the flasks were incubated an additional 72 h in dried condition in order to simulate natural situation. To stain mature biofilm, bacteria were collected by adding 50 μL of sterile distilled water (SDW) and scraping them from the bottom of the flask followed by the staining procedures described above.

To detect eDNA in exponential growth phase bacteria, an aliquot of 50 μL was collected from LB liquid cultures of *Xcc* 306 and *Xcc* 12879 A^w^ and deposited onto a glass slide following the staining procedures described above.

To detect eDNA in plate growth *Xcc* 306 and *Xcc* 12879 A^w^ carrying the GFP plasmid pUFZ75 [3,42] were collected from LB+K, stained with propidium iodide from the Live/Dead Bacterial viability kit (Molecular Probes Europe BV; Leiden, The Netherlands), deposited on a glass slide and observed with fluorescence microscopy.

To evaluate twitching motility, bacteria in exponential growth phase were washed twice with 10 mM MgCl_2_ and suspended in 1.0 mL of MgCl_2_ in a 1.5 mL centrifuge tube. Bacteria were then centrifuged at 10,000 g for 10 min and the supernatant was discarded. Bacteria were inoculated with a toothpick in PYM agar medium (Peptone 0.5%, Yeast extract 0.3%, Malt extract 0.3% and bacteriological agar 1%) supplemented with 2% glucose. Plates were incubated at 27°C for 7 d. In order to estimate the twitching production and observe the twitching halo the medium was removed and plates were washed and stained with 0.3% of CV. To observe extracellular structures and eDNA the twitching colony was stained with SYTO-9 or CV. After 20 min of staining, plates were washed with SDW and observed under the light and fluorescence microscope.

### Determination of eDNA in biofilms

To confirm the presence of eDNA in biofilm, DNAse (Deoxyribonuclease I from bovine pancreas (2000 units mg^-1^); Sigma Aldrich Inc., St. Louis, MO, USA) was added at different concentrations to static cultures of *Xanthomonas* strains at different times.

In order to visualize the effect of DNAse in the extracellular matrix, bacterial broth cultures were treated with DNAse at a 40 Kunitz mL^-1^ concentration and observed by transmission electron microscopy (TEM) after staining with 1% uranyl acetate.

To evaluate biofilm formation, bacterial cultures in exponential growth phase, performed as described above, were washed twice with 10 mM MgCl_2_ and suspended at 10^8^ cfu mL^-1^ in XVM2 medium. XVM2 medium has been demonstrated to increase biofilm formation for *Xcc* strains when compared to other media such as LB [31,42]. Microtiter plate wells were filled with 200 μL of the bacterial culture and incubated without shaking for 72 h at 27°C. After removing the medium, bacteria were incubated for an additional 72 h in dried conditions, as described above for staining mature biofilms. Biofilm formation was measured after rinsing the plates with SDW and staining with 0.3% CV for 15 min. Excess stain was removed by rinsing the plates with SDW. Residual CV in each well was solubilized in 200μL of an acetone (20%), ethanol (80%) mixture and the absorption of the extract measured in a microtiter plate reader set at A570 nm wavelength. Absorption values for each strain and treatment were calculated as the mean of readings from three wells from at least three different assays. The means were compared by analysis of variance (ANOVA) and separated by Student-Newman-Keuls (SNK) multiple range test using Statgraphics Plus for Windows 4.1 (Statistical Graphics, Rockville, MD).

For the different treatments, DNAse I was suspended in 0.15 M of NaCl and then in DNAse I reaction buffer (10mM of MgCl_2_ and 50% of glycerol suspended in 10mM of Tris-HCl at pH 7.5) as previously described [7]. DNAse I was added at concentrations of 20 or 40 Kunitz mL^-1^ at 0, 24, 48 and 72 hps to evaluate eDNA effect on biofilm development or on preformed biofilm. In order to determine if variation in biofilm was affected by the reaction buffer, a control treatment with the buffer without DNAse I was included.

## Conclusions

Presence of eDNA in *Xcc* with different host range is described as an important component of filaments connecting bacteria during bacterial growth, biofilm formation and twitching motility. In addition, fibers comprising eDNA were thicker and more connective during biofilm formation by different *Xcc* strains in certain culture media.

The role of eDNA in biofilm formation was determined for citrus and crucifer pathogenic xanthomonads. Whereas DNAse treatments did not produce a significant effect on biofilm of *Xc* 1609 strain, for citrus xanthomonads, both *Xcc* and *Xac*, biofilms were reduced after DNAse treatments performed at early stages of biofilm formation as well as in mature aggregates. Furthermore, DNAse effects differed between narrow and wide host range strains of *Xcc* in early stages of biofilm; the narrow host range strains were affected over a longer period of time. Once the biofilm was established no differences were shown between the two *Xcc* strain types. Those results demonstrate the role of eDNA for biofilm formation and maintenance for citrus xanthomonads strains, at the early stage of biofilms and as structural component.

The presence and potential importance of eDNA in biofilm formation by citrus xanthomonads opens the door for future studies of new control methods for citrus bacterial canker disease based on the disruption of biofilms or interference in their formation.

## Supporting Information

S1 Fig GFP transformed *Xcc* stained with Propidium Iodide. Representative fluorescence microscopy images of *Xcc* 306 and *Xcc* 12879 A^w^ transformed with plasmid pUFZ75 [3,42] after 48 hpi plate growth in LB medium stained with propidium iodide. eDNA fibers are shown in red and in some areas marked with white arrows.
(TIF)

S2 Fig Bacterial population after DNAse treatments. Bacterial population of xanthomonads strains was estimated in biofilm induction condition (XVM2 medium and static growth) after DNAse treatment in order to demonstrate that DNAse did not influence bacterial population and therefore biofilm formation. No differences were observed at 0, 24 or 72 hours for the treatments assayed, solely DNAse at 40 Kunitz mL^-1^ treatment showed higher population, however biofilm formation after this treatment showed the minor biofilm formation.
(TIF)
