# Supplementary figures and images for: Presence of Extracellular DNA during Biofilm Formation by Xanthomonas citri subsp. citri Strains with Different Host Range
Source: PLoS One. 2016 Jun 1;11(6):e0156695. doi: 10.1371/journal.pone.0156695 (PMC4889101; doi:10.1371/journal.pone.0156695)

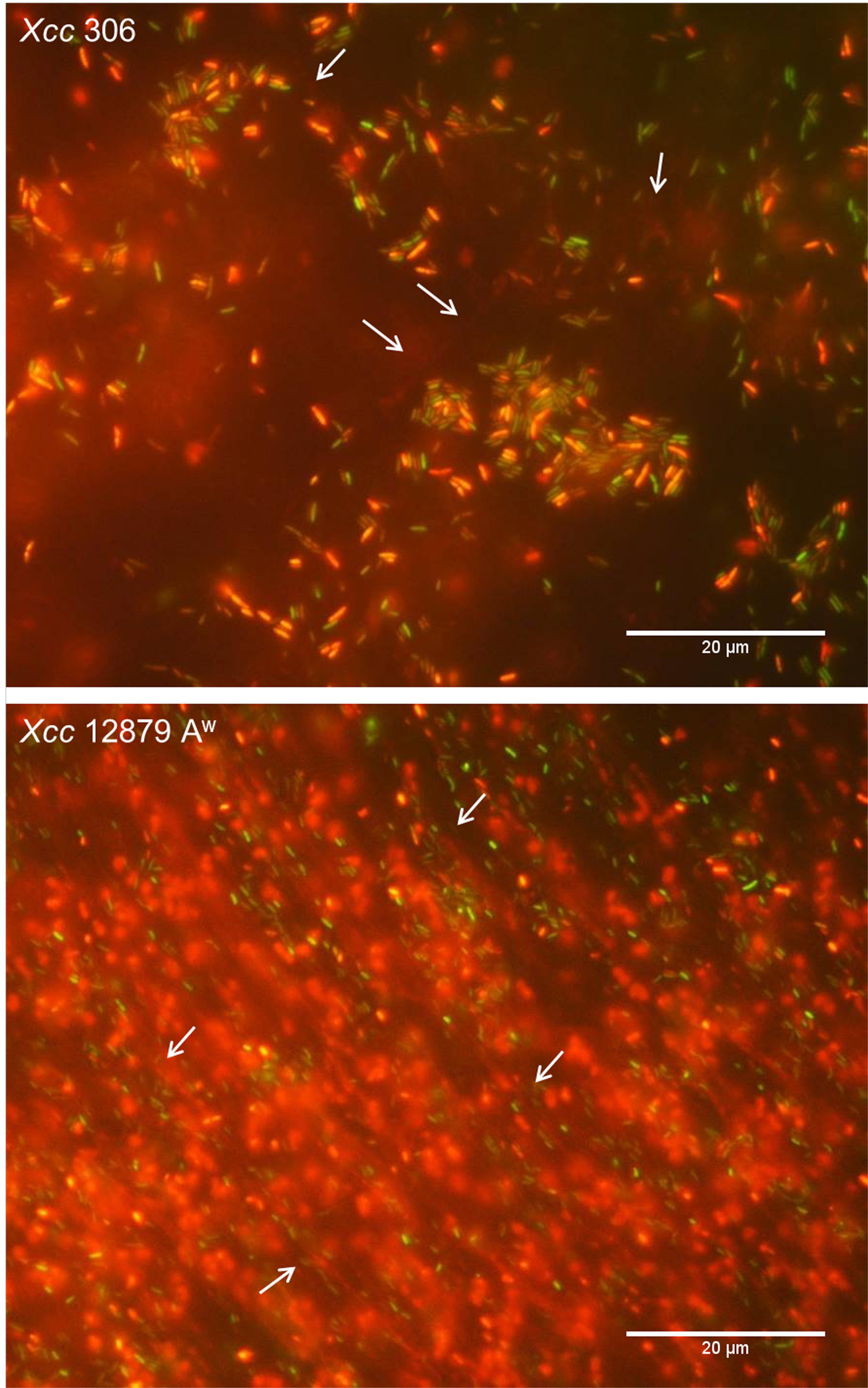

Supplement: S1 Fig — Representative fluorescence microscopy images of Xcc 306 and Xcc 12879 Aw transformed with plasmid pUFZ75 [3,42] after 48 hpi plate growth in LB medium stained with propidium iodide. eDNA fibers are shown in red and in some areas marked with white arrows. (TIF) [file pone.0156695.s001.TIF]

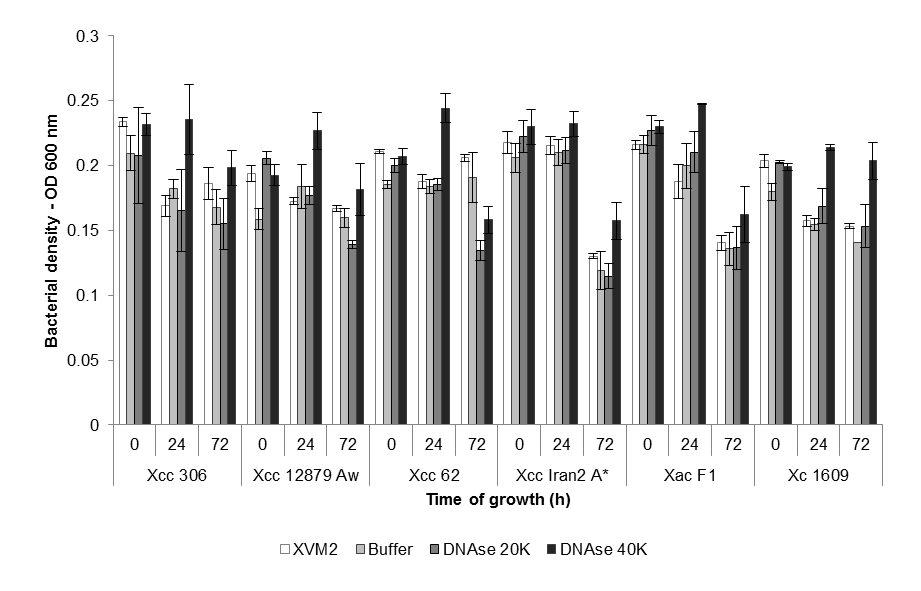

Supplement: S2 Fig — Bacterial population of xanthomonads strains was estimated in biofilm induction condition (XVM2 medium and static growth) after DNAse treatment in order to demonstrate that DNAse did not influence bacterial population and therefore biofilm formation. No differences were observed at 0, 24 or 72 hours for the treatments assayed, solely DNAse at 40 Kunitz mL-1 treatment showed higher population, however biofilm formation after this treatment showed the minor biofilm formation. (TIF) [file pone.0156695.s002.TIF]
